# Expression of core antigen of HCV genotype 3a and its evaluation as screening agent for HCV infection in Pakistan

**DOI:** 10.1186/1743-422X-8-364

**Published:** 2011-07-26

**Authors:** Muhammad Z Yousaf, Muhammad Idrees, Zafar Saleem, Irshad U Rehman, Muhammad Ali

**Affiliations:** 1 Division of Molecular Virology & Molecular Diagnostics, National Centre of Excellence in Molecular Biology, University of the Punjab, Lahore, 53700, Pakistan

## Abstract

**Background:**

Pakistan is facing a threat from hepatitis C infection which is increasing at an alarming rate throughout the country. More specific and sensitive screening assays are needed to timely and correctly diagnose this infection.

**Methods:**

After RNA extraction from specimen (HCV-3a), cDNA was synthesized that was used to amplify full length core gene of HCV 3a. After verification through PCR, DNA sequencing and BLAST, a properly oriented positive recombinant plasmid for core gene was digested with proper restriction enzymes to release the target gene which was then inserted downstream of GST encoding DNA in the same open reading frame at proper restriction sites in multiple cloning site of pGEX4t2 expression vector. Recombinant expression vector for each gene was transformed in *E. coli *BL21 (DE3) and induced with IPTG for recombinant fusion protein production that was then purified through affinity chromatography. Western blot and Enzyme Linked Immunosorbant Assay (ELISA) were used to detect immuno-reactivity of the recombinant protein.

**Results:**

The HCV core antigen produced in prokaryotic expression system was reactive and used to develop a screening assay. After validating the positivity (100%) and negativity (100%) of in-house anti-HCV screening assay through a standardized panel of 200 HCV positive and 200 HCV negative sera, a group of 120 serum specimens of suspected HCV infection were subjected to comparative analysis of our method with commercially available assay. The comparison confirmed that our method is more specific than the commercially available assays for HCV strains circulating in this specific geographical region of the world and could thus be used for HCV screening in Pakistan.

**Conclusion:**

In this study, we devised a screening assay after successful PCR amplification, isolation, sequencing, expression and purification of core antigen of HCV genotype 3a. Our developed screening assay is more sensitive, specific and reproducible than the commercially available screening assays in Pakistan.

## Background

Hepatitis C is one of the most common liver diseases around the world. It is caused by hepatitis C virus (HCV) and a significant number of patients progress towards chronic hepatitis, hepatocellular carcinoma (HCC) and liver cirrhosis [1]. Viral infection is the major cause of liver cirrhosis in about 20% of patients that after 10 years lead to HCC in 3% of these patients per year [2]. The prevalence of HCV infection in various locations around the world ranges from 0.5 to 10% [3]. Currently, almost 200 million people of the world population are infected with HCV [4]. HCV genotypes and many subtypes have been identified and are generally studied for epidemiology, molecular diagnosis, development of vaccines, and clinical management of the infection [5]. Still no vaccine is available and the standard treatment is neither economical nor fully effective in all the patients [6].

HCV is a positive single stranded RNA virus (*Flaviviridae *family) [7,8] that is nearly 9.6 Kb in length having a 5' non-coding region (5'NCR), a single open reading frame (ORF) encoding a polyprotein of about 3,000 amino acids and a non-coding region at 3' end (3'NCR). The HCV polyprotein is postranslationally cleaved into at least 3 structural (Core, E1 and E2) and 7 non-structural (NS2, NS3, NS4A, NS4B, NS5A, and NS5B) proteins [9,10] and these proteins play important roles in virus entry, replication, assembly, and pathogenesis through host peptidase and viral protease activities [11].

Core gene is one of the most conserved regions of HCV genome, involved in detection, quantitation [12] and genotyping [13,14]. It also interact with the envelop protein (E1) and thus forms the HCV capsid [15]. The core antigen-based assays has been reported to be helpful for the measurement of HCV RNA among the patients undergoing dialysis [16] and shown to be useful indicator for HCV viremia in asymptomatic carriers [17]. It has also been reported that the HCV core antigen-based methods aree useful for the quantitative measurement of HCV with respect to rapidness, easiness and low cost [12]. Moreover, HCV core antigen-based assay can identify up to 94% of viraemic donations given during the seronegative window phase of infection. The performance of the assay appears to be suitable for large-scale screening of blood donations [18].

To combat and timely diagnose HCV, community based serologic screening is of extreme significance due to dodgy trend of asymptomatic nature of the HCV infection [18]. For this purpose rapid, economical, sensitive and more specific assays are needed. The present work involved an effort to design such an assay using purified HCV core antigen from local isolates and to check out the opportunity of these cloned HCV core gene to be further employed in the possibility of vaccine development. We also describe the application of recombinant HCV core antigen from local isolates to formulate more specific screening assay for Pakistani population where HCV is becoming a big health problem.

## Methods

### PCR amplification, TA cloning and characterization of HCV Core gene

The viral RNA was first reverse transcribed and then used as template for polymerase chain reaction (PCR) [19]. Full-length HCV Core gene (573-bp) [GenBank: EU435145] was amplified with the following primers: the forward primer (Core-F) 5'-GGATCCTGCAAC*ATG*AGCACACTTCC -3' containing the BamHI restriction site and the reversed primer (Core-R) 5'-CTCGAGAGACGTGCCCGCCACTCT -3' containing the XhoI restriction site. The gel purified PCR product was then ligated with T4 DNA ligase to yield the constructs. The constructs were transformed into *E. coli*, and the selected bacterial transformants were verified by restriction enzyme analysis, colony PCR and sequencing.

### Homology search

The DNA sequences of the core gene of HCV 3a obtained were searched for homology with other sequences in GenBank data base using blastn, at http://www.ncbi.nlm.nih.gov/BLAST/. Different clones constructed in present study were aligned with the representative HCV core gene sequences in the GenBank database using Multalign software package. Pair wise comparisons were performed to determine percent nucleotide homology.

### Sub-cloning and construction of recombinant expression vector

Recombinant TA vector containing core cDNA of HCV genotype 3a and expression vector pGEX4t2 (Invitrogen) were digested with same restriction enzymes and ligated by T4 DNA ligase (Fermentas-Life Sciences, USA) overnight at 14°C, and stored at -20°C. The ligation product was routinely transformed into *Escherichia coli *(*E. coli *) DH5α by heat shock method and selected on LB broth containing ampicilline (100 ug/ml). Then the recombinant expression vector carrying target gene (pGEX4t2-C) was transformed into *E. coli *BL21 (DE3) resulting in the production of GST-C fusion protein.

### Expression and purification

The positive individual clone was cultured in 5 mL Luria bertani (LB) medium containing 100 μg/mL ampicilline and then induced for 4 hours at 37°C, adding isopropyl-β-D-thiogalactoside (IPTG) at concentration of 1 mM. For IPTG dose optimization, the bacterial culture was induced with different concentrations of IPTG [0.2, 0.3, 0.4, 0.5, 0.6, 0.7, 0.8, 0.9, 1.0 (mM)] and allowed to grow for 4 h at 37°C. For temperature optimization, the bacterial culture was induced with IPTG [1.0 (mM)] and allowed to grow for 4 h at three different temperatures (25, 30 and 37°C). For time optimization, the bacterial culture was induced with IPTG [1.0 (mM)] and allowed to grow for 3 h, 4 h, 5 h and overnight (~16 h) at 37°C. Total cell proteins from each optimization experiment were analyzed by sodium dodecyl sulfate-polyacrylamide gel electrophoresis (SDS-PAGE). Then, small-scale expression was done by optimized conditions as described above to prepare for purification [20]. The protein amount was determined by Bradford assay using bovine serum albumin (BSA) as previously described [21].

The supernatant was then poured on to a purification column and allowed to bind for 1 h with gentle shaking. Finally, the proteins were collected and analyzed by SDS-PAGE to assess the level of homogeneity. A 500 ml induced bacterial culture (*E*. coli BL21 (DE3) was harvested after 4 h, centrifuged at 6000 × g for 10 min and the cell pellet was suspended in 20 mM Tris buffer (pH 8.0). Different conditions like suitable liquid growth media for *E. coli*, suitable growth temperature and pH, IPTG final concentration for induction, post-induction time for maximum recombinant production, were optimized. The cells were later lysed using lysozyme (0.1 mg/mL) at 4°C for 1 h and sonicated on ice for 5 min at an amplitude of 30% with a 30 s pulse frequency. The lysate was centrifuged at 10,000 × g for 20 min at 4°C and the supernatant was collected as soluble fraction. The resulting pellet was washed twice with 10 mL 2M urea containing 50 mM Tris buffer (pH = 8.0), 1 mM EDTA, 150 mM NaCl and 0.1% Triton X-100. The suspension was centrifuged at 10,000 × g for 20 min at 4°C and then the resulting subsidence was resuspended in regeneration buffer containing 6 M urea, 0.5 M NaCl, 20 mM Tris-HCl (pH 7.9) and incubated at room temperature for 30 min. The incubated mixtures were then centrifuged at 10,000 × g for 20 min, and the supernatant was submitted to further purification.

After washing the column twice with 1X PBS, the clear cell free lysate was loaded directly on GST-Fast Flow Sepharose column (Amersham) with speed of 0.5 ml/min. Elution of the GST fusion protein complex was done with 10 mM reduced glutathione prepared in 15 mM Tris-Cl (pH 8). Eluted fusion protein was dialyzed in buffer containing 50 mM Tris-Cl pH 8 and 1 mM EDTA for 4 hours. Peak fractions were analyzed by western blot and ELISA.

### Western blot

Western blot analysis of samples was carried out as described previously [22]. In short, the proteins were separated on a 10-12% separating gel and transferred to nitrocellulose membrane electrophoretically. To prevent non-specific binding, the membrane was blocked in PBST (containing 0.5% Tween 20 and 2% BSA) blocking buffer. Recombinant fusion protein was identified by using rabbit polyclonal anti-GST antibody (Sigma) as primary antibody and anti-rabbit horse radish peroxidase conjugated antibody (Sigma) as secondary antibody.

### Indirect ELISA

Enzyme linked immunosorbant assay (ELISA) was performed by modifying the methods described previously [23,24] (Engvall and Perlman, 1971; Lin et al. 2008). All the values were represented by mean and standard deviation (SD) as performance of the serological assays is reported to be improved by adding 3 to 4 times the SD to the mean OD value. For the comparison and analysis of different ELISA assays SD and coefficient of variation (CV%) were used where needed.

### HCV RNA PCR

HCV RNA qualitative PCR was done using SmartCyclerII Real-time PCR with kits from Sacace Biotechnologies, Italy as per procedure given in kit protocol.

## Results

### Expression and purification of recombinant core HCV 3a antigen

**Figure 1:**
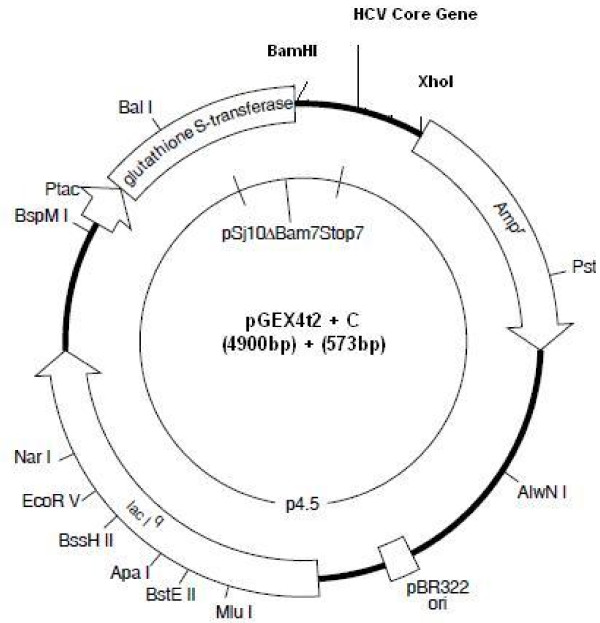
**Strategy of vector construction**. (A) Scheme of the arrangement of HCV core gene in the expression vector pGEX4t2 that leads to the construction of recombinant vector pGEX4t2C.

**Figure 2:**
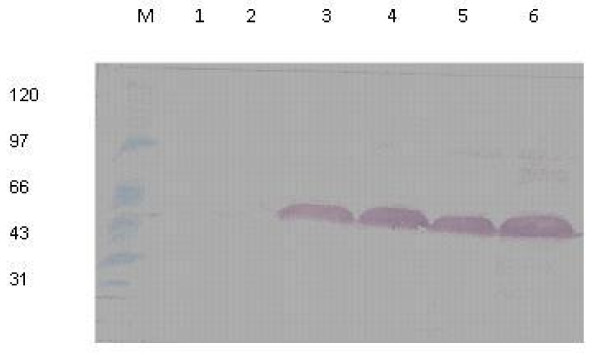
**Western blot analysis of recombinant GST+C proteins fusion complex**. Lane M refers to the resolved pre-stained protein marker, lanes 1- 2 to cell lysate of non-induced *E. coli *BL21 (DE3) cells while lanes 3-6 refer to fusion complex of GST+E1 recombinant proteins with a specific band of 46 kDa.

The cloning strategy adapted for constructing the recombinant plasmids is revealed in Figure 1. The N-terminally GST-fused recombinant core protein was produced in *E. coli *strain BL21 (DE3) with molecular weight of about 46 kDa (Figure 2). The highest level of expression was achieved in 2 × YT growth media (pH 7.5) at 25°C, with a 3 hours post-induction time, using 0.5 mM IPTG as an inducer. The strategy of our research showed more promising results than other eukaryotic systems or insect cell system because of its less time consuming, high-level yield, convenience and cost-effective. Finally, after the optimization of all conditions for expression, we obtained 8.25 mg/500 ml of protein from GST + Core construct.

### Validation of recombinant core antigen as screening agent

A standardized panel, established by the Division of Molecular Virology, Center of Excellence in Molecular Biology, consisting of 200 HCV negative and 200 HCV positive specimens were tested by ELISA using the recombinant structural core HCV 3a fusion protein and the anti-human IgG conjugate. The results obtained after calculating the means, standard deviations and co-efficient of variation (CV %). Positive and negative results were differentiated on the basis of cut-off value, which was calculated from the arithmetic mean of sera negative for HCV infection plus four randomly chosen standard deviations. Based on this cut-off value (= 0.21), the results of anti-HCV screening assay were calculated as described previously [25]. Based on this value, all positive samples were found positive and all negative samples were found negative. Validating in-house anti-HCV screening assay via known sera, 100% positivity and negativity respectively were achieved.

In this connection, the present assay was analyzed by classifying the HCV-positive and HCV-negative samples into three groups each, and having them assayed three times by the same person or by three different persons. The coefficient of variations obtained from the intra-person reproducibility for HCV positive and negative sera were 4.88% and 5.88% respectively as shown in Table 1. After validating and establishing the reproducibility (Table 2) of present in-house anti-HCV screening assay with known positive and negative sera, a total of 120 serum specimens of unknown results for HCV infection were randomly collected representing almost all geographical regions of Pakistan.

**Table 1:** Results of in-house anti-HCV screening assay

	Mean	Minimum	Maximum	**S.D**.	CV%
**Positive (n = 200)**	0.82	0.58	0.91	0.051	6.21

**Negative (n = 200)**	0.17	0.09	0.18	0.012	6.66

Number of HCV positive and negative sera was 200 each. Cut-off value was calculated 0.21. Samples exhibiting O.D. greater than 0.21 were considered positive while those exhibiting less than 0.21 considered negative. Lower the value of Coefficient of Variation (CV%) more authentic the results. Statistically, CV% of maximum 10 is acceptable for the validation of the results. Coefficient of Variation percentage is mostly affected by the extreme values in the data.

**Table 2:** Intra-person and inter-person reproducibility of in-house anti-HCV screening assay

	Intra-person reproducibility		Inter-person reproducibility	
**Anti-HCV**	Mean OD ± S.D	CV (%)	Mean OD ± S.D.	CV (%)

**Positive (n = 200)**	0.860 ± 0.042	4.88	0.859 ± 0.051	5.93

**Negative (n = 200)**	0.17 ± 0.010	5.88	0.160 ± 0.011	6.87

Mean optical densities (OD), standard deviations (SD) and co-efficient of variations (CV) of an established panel of HCV positive and negative sera are shown depicting high reproducibity.

To detect HCV infection, samples were subjected to a commercially available ELISA assay (following the manufacturer's protocol), ELISA assay validated in this study and reverse transcriptase PCR (as a 'Gold standard'). Comparative analysis of unknown sera by these three assays is summarized in Table 3. Out of total 120 sera, 36 sera were positive by both our assay (sensitivity 100%) and PCR but one sample out of these 36 was found negative by the commercial assay (sensitivity 97.2%).

**Table 3:** Comparative analysis of in-house screening ELISA assay, commercially available ELISA assay and HCV RNA PCR as reference standard

	Core antigen assay	Commercial assay	PCR 'Gold Standard'
**HCV true positive**	36 (100%)	35 (97.2%)	36

**HCV true negative**	83 (98.8%)	80 (95.2%)	84

**HCV false positive**	0 (0%)	1 (2.88%)	0

**HCV false negative**	1 (1.2%)	4 (4.76%)	0

**Total**	120	120	120

Sensitivity of our assay and commercial assay was 100 and 97.2%, while specificity was 98.8 and 95.2% respectively. The HCV RNA PCR was used as a standard reference in the comparison.

Remaining 84 sera were negative by PCR, out of these 84 negative samples, our assay confirmed 83 (98.8%) as negative but commercial assay gave false negative results for 4 samples. It is demonstrated that in-house anti-HCV screening assay has a high sensitivity, specificity and reproducibility for detection of anti-HCV antibodies.

## Discussion

Screening and diagnosing HCV is a key factor to treat this infection as early as possible and to ensure the recovery before it gets lethal. HCV infection is spreading at an alarming rate in Pakistan and 10% of the population is already HCV infected and the rate is still ascending [26]. Imported kits are used for the screening of HCV in Pakistan, however, these assays are associated with two main concerns. First, the kits are not prepared from the antigens representing local HCV strain owing to this a considerable percentage of the suspected individuals are being given false results especially false negative. Secondly, Pakistan is poor country and cannot afford precious foreign exchange that is being utilized as a result of importing these HCV screening kits. Moreover, in routine healthy blood donors screening at blood collection centers of developing countries like Pakistan, rapid assay kit to screen HCV is erroneous because of misleading results [27]. The asymptomatic nature of HCV 'The Silent Killer' [28] is posing a serious threat to Pakistani society.

In this study, we did isolate, cloned, characterized, expressed and purified the full length core antigen of Pakistani hepatitis C virus genotype 3a. The *in vitro *antigenic activity of purified recombinant proteins paved the way to develop an in-house anti-HCV screening assay for the most prevalent HCV genotype (3a) in Pakistan. The main aim of our study was to evaluate core antigen as anti-HCV screening agent and for that purpose *E. coli *expression system was convenient. Heterogeneously expressed proteins in *E. coli *are widely used for the development of screening assays for a number of diseases including HCV infection. Our research findings shows more promised results than other eukaryotic systems and insect cell system as it is more effective, produces relatively high-level yield and convenient. Further studies are needed, including elucidation of more characteristics of the recombinant structural fusion proteins and detection of anti-HCV antibodies in human sera on large scale.

In several studies HCV core antigen based ELISA has been used as a screening method for the identification of HCV infection in human sera [29-31]. No screening method has been developed so far in Pakistan based on HCV types and isolates exist in this region and the sensitivity and specificity of the commercially available methods may be low for HCV strains common in Pakistan. Keeping in view these limitations of the available assays, anti-HCV screening assay based on local HCV isolates was developed and validated using recombinant core antigen purified in the present study.

The HCV core antigen was first tested using 200 serum samples with established HCV infection cases and 200 serum samples from anti-HCV and HCV RNA negative sera to evaluate the sensitivity and specificity respectively of the developed method. Detection of HCV RNA through RealTime PCR was used as a reference to compare and validate the results. The HCV core antigen and the reference tests detected all 200 HCV positive samples as positive and all 200 negative samples as negative. The results are in accordance with the previous reports. A report from Iran [32] described the expression of HCV core antigen in *E. coli *but instead of ELISA, dot blot assay was preferred to capture the antibodies in HCV infected human sera. Important factors for a commercial assay are its specificity and reproducibility. An assay must yield concordant results when tests are repeated [33]. In the present study, the assay was analyzed by classifying the HCV-positive and negative sera and concluded that the reproducibility was sufficiently high.

To avoid false results, we used core antigen of HCV for the most prevalent genotype (3a) in Pakistan. As compared to ELISA, the rapid tests have not shown any promising results and hence should not be recommended in transfusion centers for screening blood donors. Moreover, the failure of the rapid kits to detect HCV reactive samples may be due to inadequate coating of the antigens, heterogeneity of the virus nature of the antigens used [34]. These evaluations focused on the working characteristics of the present in house anti-HCV screening assay, such as ease of handling, specificity and sensitivity on a group of well-characterized samples obtained from geographically diverse regions of Pakistan, and we report their suitability for manipulation in small laboratories, *i.e*. blood collection centers. A possible drawback of the present HCV core antigen based ELISA is that it may not detect HCV infection in the sample taken prior to the production of anti-HCV antibodies in the patients i.e. the period between actual infection and production of antibodies.

## Conclusions

In the present study, we were able to obtain a high-level expression of the recombinant HCV core antigen. In this study, we devised a screening assay by using core antigen of local HCV genotype 3a. This is the first report of its own kind in which the core antigen of HCV from a local strain was successfully used as screening agent. The sensitivity, specificity and reproducibility of the developed assay is high than the commercially available ELISA assays.

## Competing interests

The authors declare that they have no competing interests.

## Authors' contributions

MI and MZY conceived the study. MZY collected the samples and performed the molecular analysis. ZS and IR helped MZY in performing the work. MZY searched the literature and drafted the manuscript. MI, MA and IR critically reviewed the manuscript. All the authors read and approved the final manuscript.

## Sources of support

This work was partially supported by the Higher Education Commission (HEC) of Pakistan.
